# Systematic development of a theory-informed multifaceted behavioural intervention to increase physical activity of adults with type 2 diabetes in routine primary care: Movement as Medicine for Type 2 Diabetes

**DOI:** 10.1186/s13012-016-0459-6

**Published:** 2016-07-19

**Authors:** Leah Avery, Sarah J. Charman, Louise Taylor, Darren Flynn, Kylie Mosely, Jane Speight, Matthew Lievesley, Roy Taylor, Falko F. Sniehotta, Michael I. Trenell

**Affiliations:** 1 Institute of Cellular Medicine, Faculty of Medical Sciences, Newcastle University, Newcastle upon Tyne, NE2 4HH UK; 2 Institute of Health & Society, Newcastle University, Newcastle upon Tyne, NE2 4AX UK; 3 Graduate School of Health, University of Technology, Sydney, New South Wales 2007 Australia; 4 AHP Research Limited, Hornchurch, Essex UK; 5 The Australian Centre for Behavioural Research in Diabetes, Diabetes Victoria, Melbourne, Victoria 3000 Australia; 6 School of Psychology, Deakin University, Victoria, 3125 Australia; 7 Northumbria School of Design, Northumbria University, Newcastle upon Tyne, NE1 8ST UK

**Keywords:** Behavioural intervention, Healthcare professional behaviour change, Type 2 diabetes, Physical activity, Primary care

## Abstract

**Background:**

Despite substantial evidence for physical activity (PA) as a management option for type 2 diabetes, there remains a lack of PA behavioural interventions suitable for delivery in primary care. This paper describes the systematic development of an evidence-informed PA behavioural intervention for use during routine primary care consultations.

**Methods:**

In accordance with the Medical Research Council Framework for the Development and Evaluation of Complex Interventions, a four-stage systematic development process was undertaken: (1) exploratory work involving interviews and workshop discussions identified training needs of healthcare professionals and support needs of adults with type 2 diabetes; (2) a systematic review with meta- and moderator analyses identified behaviour change techniques and optimal intervention intensity and duration; (3) usability testing identified strategies to increase implementation of the intervention in primary care and (4) an open pilot study in two primary care practices facilitated intervention optimisation.

**Results:**

Healthcare professional training needs included knowledge about type, intensity and duration of PA sufficient to improve glycaemic control and acquisition of skills to promote PA behaviour change. Patients lacked knowledge about type 2 diabetes and skills to enable them to make sustainable changes to their level of PA. An accredited online training programme for healthcare professionals and a professional-delivered behavioural intervention for adults with type 2 diabetes were subsequently developed. This multifaceted intervention was informed by the theory of planned behaviour and social cognitive theory and consisted of 15 behaviour change techniques. Intervention intensity and duration were informed by a systematic review. Usability testing resolved technical problems with the online training intervention that facilitated use on practice IT systems. An open pilot study of the intervention with fidelity of delivery assessment informed optimisation and identified mechanisms to enhance implementation of the intervention during routine diabetes consultations.

**Conclusions:**

Movement as Medicine for Type 2 diabetes represents an evidence-informed multifaceted behavioural intervention targeting PA for management of type 2 diabetes developed for delivery in primary care. The structured development process undertaken enhances transparency of intervention content, replicability and scalability. Movement as Medicine for Type 2 diabetes is currently undergoing evaluation in a pilot RCT.

**Trial registration:**

ISRCTN67997502

**Electronic supplementary material:**

The online version of this article (doi:10.1186/s13012-016-0459-6) contains supplementary material, which is available to authorized users.

## Background

Effective management of type 2 diabetes poses a significant medical, behavioural and public health challenge, particularly with the projected increases in prevalence largely attributable to an ageing population and unhealthy lifestyle behaviours [1]. Type 2 diabetes is generally regarded as a progressive condition [2]; however, a plethora of evidence has demonstrated that a range of management options, in particular lifestyle behaviour modification (e.g. physical activity and diet), can result in improved glycaemic control [3]. These in turn can decelerate, halt or even reverse the progression of type 2 diabetes and significantly reduce the risk of serious complications and premature mortality [4].

Physical activity (PA) is an effective management approach for long-term glycaemic control in adults with type 2 diabetes [5–8]. Yet, the majority of people with type 2 diabetes are physically inactive compared with national norms [9]. UK guidelines indicate the need for patient education models for the management of type 2 diabetes [10] and recommend that people with type 2 diabetes are offered some form of education/intervention at least at the point of diagnosis. However, this is not available universally and, where this is available, the length, information content and style of the interventions vary greatly between services. Despite being an important part of self-management, PA advice and concomitant interventions are frequently omitted from management plans for people with type 2 diabetes in the primary care setting [11].

As primary care is the main setting for the majority of type 2 diabetes healthcare, it is arguably the optimal setting for targeting PA where multiple opportunities exist for the delivery of effective interventions by primary healthcare professionals. However, there remains a lack of evidence-informed interventions targeting PA that can be offered to people with type 2 diabetes during routine primary care consultations. Furthermore, healthcare professionals often report difficulties in supporting their patients to increase their everyday levels of PA [12] largely due to lack of training in lifestyle behaviour change [13, 14]. With traditional clinician-led advice-giving and direct persuasion approaches being ineffective for a large proportion of patients [13], behavioural interventions that can increase PA to a degree sufficient to have a clinically significant impact on glycaemic control in the long-term are needed.

The majority of available interventions do not include adequate support to help people with type 2 diabetes to achieve PA recommendations; very few have been formally evaluated and rarely have the individuals responsible for the delivery of these interventions been formally trained for this purpose [14].

Currently available structured type 2 diabetes programmes (e.g. Diabetes Education and Self-Management for Ongoing and Diagnosed (DESMOND) and the X-PERT Diabetes Programme) have demonstrated effectiveness for increasing self-efficacy [15] and improving glycaemic control [16]. However, these programmes require referral, regularly have protracted waiting lists and are not available to primary care practices in all areas of UK. Furthermore, primary healthcare professionals do not receive training to provide follow-up support. Therefore, there remains a pressing need for an intervention that (1) is available to all patients with type 2 diabetes and is available when required (i.e. at key teachable moments within primary care appointments); (2) provides primary healthcare professionals with the skills they require to successfully target PA during routine diabetes care; (3) provides patients with the support and resources to effectively increase their levels of PA in the context of their everyday lives and (4) can be delivered within the primary care setting.

The Medical Research Council (MRC) framework for the development and evaluation of complex interventions highlights the need to clearly describe the intervention development process and describes four phases: development (identify the evidence base, identify and develop theory and model processes and outcomes); feasibility/piloting (test intervention components and processes, estimate recruitment/retention and determine sample size); evaluation (assess effectiveness and understand processes of change) and implementation (dissemination, surveillance/monitoring and long-term follow-up) [17, 18]. Behaviour change interventions are, by their nature, complex because they contain multiple components; however, details of the development process and reporting on specific details about their content are often lacking [19, 20]. This is problematic when trying to replicate interventions or when trying to identify what active ingredients contribute to their acceptability, feasibility and effectiveness. This paper describes the application of the MRC’s framework to the development of Movement as Medicine for Type 2 diabetes, a theory-informed multifaceted behavioural intervention to increase PA of adults with type 2 diabetes in routine primary care. The intervention is multifaceted as it comprises of two facets or ‘levels’. The first is an online training intervention programme that aims to provide healthcare professionals with the skills required to target PA behaviour change of their patients during consultations. The second is a set of resources (i.e. a patient DVD, pedometer and paper-based materials) designed for delivery by a healthcare professional to support patients to make changes to their PA levels.

We previously published a study protocol [21] that described the methodology for an open pilot study (phase 1) and pilot RCT (phase 2) of a multifaceted behavioural intervention. The current paper supplements the protocol with a comprehensive description of the intervention development process. This includes how the intervention content was iteratively designed with reference to patient and healthcare professional’s views and perspectives identified from the findings of exploratory work, usability testing and the mixed methods open pilot study.

## Methods

**Fig. 1:**
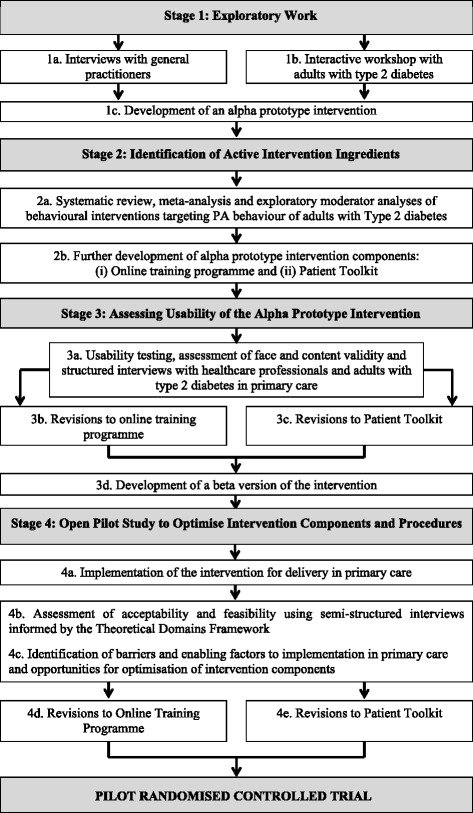
Overview of the intervention development process undertaken to develop the theory-informed multifaceted behavioural intervention

A four-stage systematic development process was undertaken (see Fig. 1) to develop a multifaceted PA behavioural intervention with reference to three phases: pre-clinical (theoretical—exploratory work with patients and healthcare professionals), modelling (phase I—systematic review with meta- and moderator analyses and usability testing of an alpha prototype with patients and healthcare professionals) and exploratory trial (phase II—mixed methods open pilot study with fidelity assessment). The aim was to develop a behaviour change intervention that (1) fulfilled the needs of both primary healthcare professionals and adults with type 2 diabetes; (2) was evidence- and theory-informed; (3) could be successfully integrated into routine primary care and (4) could be appropriately evaluated.

### Stage 1: exploratory work

The need for a multifaceted behavioural intervention originated from a local Primary Care Trust that had identified training for primary healthcare professionals on PA behaviour change as a priority area. Therefore, key informant general practitioners (GPs) from practices within the Trust area and patients were initially consulted by engaging them in a co-development process. The aim was to explore the views and experiences of each group to inform the content and mode of delivery of an intervention designed to effectively target PA in the primary care setting.

#### Exploratory work with primary healthcare professionals

A purposive sample of four GPs from four primary care practices in North East England participated in a semi-structured interview to gain in-depth information from key informants (three were specialists in diabetes and all four took overall responsibility for diabetes care and training provision within their primary care practices) on current practices and needs related to a more effective delivery of PA interventions. A topic guide was used to elicit information on how patients from each practice typically received their diabetes education and advice on PA (at what time points following diagnosis); to what extent participating GPs believed patients engaged with the process of diabetes supported self-management; approaches they currently use to target PA during diabetes review clinics and their views on whether they felt they were effective. Information was also obtained on their preferences for mode of training and intervention delivery and the amount of time available to complete training within a typical working week. All interviews were audio recorded, transcribed verbatim and analysed using content analysis [22].

##### Key findings of exploratory work with GPs

In general, prescribing medication was considered a more attractive and effective option than promoting PA behaviour change for adults with type 2 diabetes. Lifestyle behaviour change was considered to be the most difficult part of diabetes management, with GPs reliant on advice-giving and limited to providing specific suggestions to patients in terms of activity type and frequency (e.g. walking for 30 min three times per week).

GPs were knowledgeable about the underlying physiological mechanisms of type 2 diabetes but frequently experienced difficulty with communicating this complex information to patients. Furthermore, they expressed dissatisfaction that many of their patients do not act upon their advice about increasing their PA levels. As such, they stated that a different approach was desirable to effectively communicate information about diabetes to patients, including the benefits of leading a physically active lifestyle that would be more amenable to patients’ personal situations. They reported patients typically adopting a passive role during consultations, with a reluctance to engage in discussions about self-management of their diabetes and other comorbidities by changing their lifestyle behaviour. The latter was interpreted by GPs as patients failing to take responsibility*.* Structured diabetes education at the point of diagnosis was offered to patients often by referral to a programme such as *Diabetes Education and Self-Management for Ongoing and Newly Diagnosed* (DESMOND) or X-Pert [15, 16]. However, GPs indicated that many of their patients preferred not to take advantage of this service and also frequently did not attend scheduled diabetes review appointments.

Important considerations emerged in the context of GPs’ training needs, including being able to plan a programme of training in advance to feed into the annual appraisal of practice for GPs, and for the training to be evidence-based and accredited for the purposes of continuing professional development. Time for training was described as ‘limited’, although an online programme that allowed healthcare professionals to work through the content on multiple occasions over a specified period of time was considered to be a feasible mode of training delivery. The importance of being able to identify a patient’s current level of PA was also emphasised (i.e. the need for tools to achieve this) as a starting point for discussion about PA behaviour change.

Exploratory work identified a need for a training programme designed to increase healthcare professionals’ awareness of the value of PA as a management option for type 2 diabetes (i.e. it was reported that diet was targeted more consistently than PA due to the lack of knowledge about the type, duration and frequency of PA to recommend to patients). Furthermore, the focus of the training should be on skills development in the context of PA for type 2 diabetes utilising evidence-based health behaviour change strategies. There was consensus amongst the GPs interviewed that an online training programme would allow flexibility and demonstrate new ways to communicate to patients about diabetes, in particular, why it progresses without appropriate management and how patients can self-manage their diabetes effectively by increasing their PA behaviour.

The GPs who participated in the initial exploratory work did not take part in the subsequent developmental stages as we wanted to obtain views and feedback from a range of healthcare professionals throughout the development process.

#### Exploratory work with adult patients with type 2 diabetes

An interactive workshop involving six adults with type 2 diabetes aged ≥65 years with a median time since diagnosis of 11 years (interquartile range (IQR) = 8) was facilitated by two of the authors (LT and ML) to gain insights into their lived experience of diabetes, as well as identifying areas of care delivery from their perspective to consider for inclusion in the facet of the intervention for primary healthcare professionals. The benefits of the group-based workshops were that participants could ask the group questions and facilitators could immediately clarify any queries and provide feedback in order gain an in-depth understanding of the multiplicity of thoughts, feelings and views about a specific topic, which were more likely to be elicited in a social setting involving direct interaction. Therefore, the workshop was used as a vehicle to explore patients’ views and perspectives on the following diabetes-related topics: the value of the information provided by primary healthcare professionals on diabetes; support needs to effectively manage their diabetes by increasing PA levels; whether they believed a family history of diabetes influenced their knowledge about the condition and the role of lifestyle behaviour change; who they would contact to request information and support about type 2 diabetes when required; how well informed they were about the use of PA as a management option for their diabetes and how much information and support had they had received previously to increase their everyday levels of PA and whether they felt this was beneficial.

Following the initial group discussions, patients were asked to complete a ‘My Diabetes Story’ worksheet (see Additional file 1) designed to elicit reflection on the high and low points of living with type 2 diabetes, and a ‘Circle of Influence’ worksheet (see Additional file 2) used to map the most influential people (healthcare professionals and others) in the context of their diabetes care. The completed worksheets were subsequently used as prompts for group discussion on the challenges of living with and successfully managing type 2 diabetes, as well as their experiences of receiving diabetes care from primary healthcare professionals. Finally, participants were asked to complete a worksheet exercise designed to identify the key characteristics of a good health professional (see Additional file 3).

Given the varied nature of the interactive workshop (i.e. a series of structured exercises as well as verbal feedback), the session was not audio recorded. Instead, one of the two facilitators (LT) conducting the session made detailed field notes. Responses from the structured exercises and field notes on salient points were subjected to content analysis by the two workshop facilitators.

##### Key findings of exploratory work with adults with type 2 diabetes

Patients reported feeling that the primary healthcare professionals they had initial contact with following their diagnosis were not particularly knowledgeable about type 2 diabetes. As such, they felt unable to discuss their condition in any significant depth; for example, one patient stated that a practice nurse was unsure about what represented a normal HbA1c level. Patients reported educating themselves, their GP or a practice nurse about their condition and effective self-management strategies. One patient felt that secondary care provided a better service in terms of the level of support provided for their diabetes management.

A variety of experiences when receiving a diagnosis of type 2 diabetes was described by patients. A common scenario was that GPs would refer to the possibility of diabetes without explaining the condition or the implications it may have for the patient. Subsequently, participants were referred to a different primary healthcare professional for confirmation. Participants expressed the opinion that diabetes appeared to be a low priority for their primary care practice. One factor contributing to this belief was that diabetes review clinics were generally nurse-led, as opposed to GP-led.

Five of the six patients reported being advised to ‘lose weight’, ‘change their diet’ or ‘exercise more’ although they were not offered any specific support or advice on how to achieve this in the context of their everyday lives.

When asked ‘what makes a good healthcare professional in the context of diabetes?’, participants referred consistently to healthcare professionals’ knowledge of diabetes. Some held the belief that the ‘new breed’ of younger healthcare professionals were more likely to communicate with their patients as equals compared to the so-called *old school doctors,* and this was regarded as a positive step forward.

While exploring family history and in the context of their knowledge of diabetes, patients stated that it was inevitable that they would develop the condition; although for some, the diagnosis still came as a surprise. Their expectation was that their diabetes would likely progress in the same way as their parents (or other close relatives) despite their belief that diabetes treatment had vastly improved. When asked who they would visit as a first port of call for information about their diabetes, the majority relied on friends with diabetes or information they came across in the media or press. Several patients were determined to gather as much information as they could about their diabetes, whereas others were unwilling to question the information and advice given to them in person by healthcare professionals.

There was consensus amongst the patients that they could not recall receiving any direct information about the potential of PA as a management option for type 2 diabetes from primary care teams. This is despite several patients having an awareness and knowledge of the value of increased PA or exercise for glycaemic control. When asked what advice they had been given specifically about PA, patients could not recall any specific examples (other than being advised to simply take plenty of exercise) or receiving any specific information/guidance on recommended levels, type and duration of PA, including practical hints and tips on how to successfully increase their PA levels.

Patient preferences on how they would like to receive information on diabetes-related topics (and more specifically PA as a management option for diabetes, including their preferences on mode of delivery) were for printed booklets/leaflets and a DVD (all patients reported owning a DVD player). Despite reporting in some cases their dissatisfaction with the information they had received previously in primary care settings, a strong preference was elicited from patients for face-to-face delivery by a knowledgeable and respectful primary healthcare professional.

#### Conclusions of exploratory work

The findings of the exploratory work were summarised and discussed within the research team. The exploratory work highlighted a need to focus on training provision for healthcare professionals to equip them with the knowledge and skills to target PA behaviour of their patients. An online training programme was developed in accordance with GPs stated preference for mode of delivery and the need for flexibility. Consequently, the online programme was presented in a modular format to allow flexibility of completion in small manageable sections in order to maximise successful integration within a typical primary care environment.

The training needs of primary healthcare professionals were identified in terms of different types, intensities and duration of PA/exercise on health outcomes, in particular, glycaemic control; the physiological effects of a physically active lifestyle in the context of type 2 diabetes and effective communication of this information to patients; and the utilisation of evidence-based behaviour change strategies to facilitate person-centred discussions with patients about increasing and maintaining PA behaviour. In general, healthcare professionals reported lacking confidence in discussing lifestyle issues with their patients, primarily due to frustration resulting from numerous unsuccessful past attempts. Modules on each of these topics were developed for inclusion in the online training programme.

Adults with type 2 diabetes expressed a strong preference for individual face-to-face sessions with healthcare professionals who were knowledgeable about diabetes. Paper-based resources and a DVD were identified as feasible methods for conveying information about diabetes and providing support to patients. The latter finding was attributable to participants either not having access to or being unfamiliar with how to access or navigate the Internet; although they reported familiarity and access to a DVD player.

#### Development of an alpha prototype intervention

The views and experiences of participants in the exploratory work provided an initial indication of the mode, form and information content of an alpha prototype multifaceted intervention for further refinement in subsequent stages of the co-development process: (1) an online training programme for healthcare professionals to develop their knowledge and skills and (2) a suite of paper-based patient resources for use by healthcare professionals and patients during routine diabetes review appointments. These draft resources were designed to help patients to develop knowledge and skills for using PA as a vehicle for diabetes self-management. In addition, a DVD was designed to provide information to supplement and/or reinforce key information outside of consultations. The DVD included narratives of people with type 2 diabetes who had successfully increased their levels of PA with some practical hints and tips on how this could be achieved.

The specific evidence-informed information content of the behaviour change components of the alpha prototype PA behavioural intervention were further refined in stage 2 of the development process.

### Stage 2: identification of behaviour change techniques, theoretical basis, intervention intensity and duration

Prior to embarking on the development of a new intervention, the MRC framework encourages researchers to identify what is already known about other similar interventions (e.g. their theoretical processes of change) by drawing on existing evidence. Despite the growing number of RCTs examining the effect of behaviour change interventions on PA behaviour of adults with type 2 diabetes, a systematic review examining the pooled effect size of these studies on PA behaviour and glycaemic control had not been published. Stage 2 of the intervention development process involved a systematic review and meta-analysis to assess the effectiveness of PA behaviour change interventions for long-term glycaemic control and identification of candidate behaviour change techniques and other intervention features associated with improved glycaemic control.

#### Systematic review with meta- and moderator analyses

A systematic review and meta-analysis of 17 RCTs was conducted to assess whether behavioural interventions were more effective than usual clinical care for increasing PA and improving glycaemic control in a sample of 1975 adults with type 2 diabetes [14]. In summary, we demonstrated that behavioural interventions (when compared to usual care) delivered in clinical and community settings are effective for increasing objectively assessed PA and for yielding a clinically significant improvement in long-term glycaemic control as measured by HbA1c (−0.32 %, 95 % CI = −0.44 to −0.21 %).

A pooled effect size does not provide any indication of what components (i.e. active ingredients) of the interventions are associated with effectiveness. Therefore, a series of moderator analyses of the 17 studies included in the systematic review was conducted to inform selection of behaviour change techniques (using a comprehensive and reliable taxonomy [23]) and other intervention features (e.g. mode of delivery, use of theory) associated with increases in clinically significant improvements in HbA1c (i.e. 0.3 % reduction) when present (as opposed to absent) in the studies reviewed. Studies utilised in the systematic review included both subjective and objective methods of free-living PA/exercise assessment. Moreover, it was not possible to establish the type, intensity and frequency of PA/exercise attributable to the effect sizes observed. Therefore, we made the decision to identify behaviour change techniques (BCTs) associated with clinically significant improvements in HbA1c to maximise the likelihood of increasing PA/exercise to a level sufficient to impact on glycaemic control.

#### Selection of behaviour change techniques and other intervention features

Moderator analyses identified ten behaviour change techniques for inclusion in the alpha prototype PA behaviour intervention. These were prompting generalisation of a target behaviour, use of follow-up prompts, prompt review of behavioural goals, provide information on where and when to perform PA, plan social support/social change, goal setting behaviour, time management, prompting focus on past success, barrier identification/problem-solving and provide information on the consequences of behaviour to the individual.

Other intervention features identified from moderator analyses were the inclusion of ten or more behaviour change techniques: interventions underpinned by a theory/model of behaviour change and intervention durations of ≥6 months. Contrary to expectations, moderator analyses suggested that different modes of intervention delivery, interventions utilising pedometers, interventions of greater intensity (greater to of equal to a median of 14 patient contacts) and inclusion of a supervised PA or exercise component were not associated with clinically significant improvements in HbA1c.

Following a wider appraisal of the evidence base, a further three behaviour change techniques that were shown to have a neutral effect in the moderator analyses (i.e. they had a positive association with HbA1c when both are present and absent) were included in the intervention: provide feedback on performance, relapse prevention/coping planning and rewards contingent on effort/progress made towards PA behaviour. These were included for pragmatic reasons to provide a balance of motivational and volitional behaviour change techniques to support intention formation and to promote maintenance of PA behaviour [24].

Given that moderator analyses could not test associations between different combinations of behaviour change techniques, it was considered important to include combinations of techniques with strong evidence for increasing PA from the wider research literature. Therefore, a further two behaviour change techniques, action planning and prompt self-monitoring of behaviour, were selected for inclusion in the intervention despite the overall effect on HbA1c being larger when these techniques were absent. Two published reviews reported that inclusion of self-regulatory techniques such as self-monitoring in PA behaviour change interventions improved effectiveness [25, 26]. Furthermore, PA interventions are reported to be optimised when self-monitoring is utilised and combined with at least one other self-regulatory technique [27]. A pedometer was included in the intervention (despite exploratory moderator analyses indicating that interventions including these devices were not associated with clinically significant improvements in HbA1c) to augment self-monitoring of PA behaviour (i.e. as means for participants to record their progress on activity planners) and strong evidence from the wider research that pedometers significantly increase PA behaviour [28].

Similarly, interventions utilising action planning and coping planning in combination were found to predict increases in PA [29]. Moreover, the inclusion of both action planning and coping planning at various stages of the behaviour change process (motivational and volitional) is reported to be an optimal strategy to employ when targeting PA behaviour change [30, 31].

The moderator analyses indicated that the intervention duration should be ≥6 months (i.e. patients should be supported to increase their levels of PA for at least 6 months). In the absence of explicit guidance from the systematic review findings regarding the mode of delivery of the intervention, the decision to use individual face-to-face sessions with a healthcare professional and patient was guided by the preferences expressed by patients during the exploratory phase (stage 1).

#### Theory selection

Moderator analyses suggested that interventions underpinned by a theory or model of behaviour change were associated with clinically significant improvements in HbA1c). However, no single theory emerged as potentially superior over another. Therefore, explicit selection criteria were applied to inform theory selection. These included a strong evidence base for modelling the process of intervention components and outcomes that were the focus of the intervention (i.e. changing consultation behaviour of primary healthcare professionals and increasing PA in adults with type 2 diabetes). Furthermore, exploratory work undertaken in stage 1 with primary healthcare professionals and patients identified the need to target motivational factors (e.g. attitudes/beliefs regarding the use of PA by healthcare professionals as a management option for people with type 2 diabetes) and volitional factors (e.g. self-efficacy to increase and maintain increases in PA behaviour in adults with type 2 diabetes and self-efficacy of healthcare professionals for delivering a behavioural intervention during routine primary care). Therefore, additional criteria for guiding theory selection included a theory that incorporated both motivational and volitional factors [24] and the existence of in-built constructs and/or evidence-based strategies that can be used to effectively target both motivation/intention and actual PA behaviour to support maintenance. It was also important to select a theory/theories that had readily available, reliable and valid instruments to measure the theoretical constructs and postulated relationships between the constructs to inform the evaluation of the intervention in a planned pilot RCT.

In accordance with these criteria, the theory of planned behaviour (TPB) [32] and social cognitive theory (SCT) [33] were selected to inform the development and evaluation of the intervention. Together with the theory-linked behaviour change techniques identified by the moderator analyses, the TPB and SCT provided a complimentary theoretical framework for guiding the development and subsequent evaluation of the intervention. The TPB has been extensively and successfully used to predict intentions to increase PA and actual PA behaviour [34, 35] including healthcare professional behaviour change [36]. However, the TPB does not provide explicit guidance on how theoretical constructs can be targeted within interventions using evidence-informed strategies or how to address the frequent lack of concordance between motivation/intention and action (i.e. the *intention-behaviour* gap) [37]. SCT is able to provide specific evidence-based strategies for translating motivation/intentions into action/behaviour in both healthcare professionals and patients (e.g. observational learning strategies such as modelling to support the acquisition of behaviour change skills and self-efficacy for the effective delivery of behaviour change techniques to patients). Furthermore, SCT has demonstrated utility in samples of people with diabetes when predicting PA behaviour [38].

#### Defining and mapping the alpha prototype PA behaviour intervention components

Guidance published by Davidson et al. [39] was followed to ensure that intervention characteristics were described appropriately when developing the alpha prototype PA behavioural intervention. These included intervention format, intensity, duration and information content. Findings of the exploratory work and systematic review were used collectively to inform decisions about the content and mode of delivery of the alpha prototype intervention. A taxonomy of behaviour change techniques [23] was used throughout the intervention development process to ensure that techniques selected for inclusion in the intervention were defined adequately and consistently.

##### Online training component

Consistent with the needs of primary healthcare professionals (identified in stage 1), the training programme was designed as an online resource delivered in a modular format to facilitate flexibility and accessibility. The training programme was accredited by the Royal College of Physicians with completion of the programme prompting generation of a certificate awarding three continuing professional development points. This fulfilled the GPs’ requirement for the training programme to contribute towards their annual appraisal.

The content of the online training programme addressed the knowledge and skills gaps identified during the exploratory phase (stage 1). Eight distinct but interrelated modules were developed. Table 1 presents an overview of the information content of each module and the relationships between content and associated behaviour change techniques, including their relationship with the theoretical constructs of the TPB and SCT. Module 2 presented healthcare professionals with information about study procedures and data collection that was initially provided as a paper-based protocol during the consent process. As this did not form part of the PA behaviour change intervention, it is not presented in Table 1.

**Table 1:** Components of the online training programme and their relationship to constructs within the theory of planned behaviour and social cognitive theory

Module	Form and information content	Theoretical constructs	Behaviour change techniques
Module 1: introduction to MaMT2D	Video recording of a professor of movement and metabolism introducing the programme and providing details of how and why MaMT2D was developed. Video recording of a consultant diabetologist and a diabetes specialist nurse providing an overview of why PA is important for the management of T2D	Symbolising (SCT) Attitudes and beliefs (TPB) Subjective norms (TPB)	Provide information on the consequences of behaviour in general (1)
Modules 3 (metabolism and type 2 diabetes), 4 (physical activity in the care of type 2 diabetes) and 5 (physical activity and exercise)	Evidence-based information about the role of metabolism, PA and exercise in the context of type 2 diabetes	Symbolising (SCT) Attitudes/beliefs (TPB) Forethought (SCT) Intention (TPB)	Provide information on the consequences of behaviour in general (1) Provide feedback on performance (19)
Module 6: using psychology to change physical activity behaviour	Evidence-based information on the use of psychological theory and theory-linked behaviour change techniques and counselling skills to change PA behaviour	Attitudes/beliefs (TPB) Forethought (SCT) Intention (TPB)	Provide information on the consequences of behaviour in general (1)
Module 7: using behaviour change techniques to increase physical activity behaviour	Video demonstrations of a diabetes specialist nurse demonstrating the use of behaviour change techniques and behaviour change counselling techniques in practice with a mock patient	Symbolising (SCT) Observational Learning (SCT) Perceived Behavioural Control (TPB) and Self-efficacy (SCT) Subjective norms (TPB)	Self-monitoring of behaviour (16) Provide instruction on how to perform the behaviour (21) Model/demonstrate the behaviour (22) Environmental restructuring (24) Prompt practice (26) Motivational interviewing (37) General communication skills training (39)
Module 8: screening before physical activity	Flowchart diagram demonstrating how to screen adults with T2D prior to PA/exercise	Self-regulation (SCT) Perceived behavioural control (TPB) and self-efficacy (SCT)	Provide instruction on how to perform the behaviour (21)
End of modules 3, 4 and 5 quiz questions	Provides feedback on performance	Perceived behavioural control (TPB) and self-efficacy (SCT)	Provide feedback on performance (19) Provide rewards contingent on successful behaviour (12)
Flowchart summary (crib sheet with prompts) of the protocol for use of the patient toolkit during diabetes review appointments	Prompts for healthcare professionals to use specific behaviour change skills and techniques	Symbolising (SCT) Perceived behavioural control (TPB) and self-efficacy (SCT) Intention (TPB) Self-regulation (SCT)	Teach to use prompts or cues (23)

The numbers in parentheses correspond to the BCT number in the CALO-RE taxonomy

*Abbreviations: MaMT2D* Movement as Medicine for Type 2 Diabetes, *TPB* theory of planned behaviour, *SCT* social cognitive theory, *PA* physical activity

The behaviour change techniques selected for inclusion in the patient toolkit component of the intervention were covered in detail within the content of modules 6 and 7. A series of audiovisual clips portrayed simulated interactions between a diabetes specialist nurse and a patient. These were used as a vehicle to demonstrate to healthcare professionals how to use specific behaviour change techniques to facilitate delivery of the patient toolkit.

Video clips demonstrating the use of behaviour change counselling skills based on the principles of motivational interviewing (agenda setting, use of importance and confidence rulers to engage participants in change talk, active listening and informing) [40] were incorporated into module 7. These specific counselling skills have evidence for effectively engaging patients effectively in discussions about behaviour change with healthcare professionals [41]. They also serve as effective vehicles for placing the patient at the centre of the decision-making process, and as such, maintaining their autonomy when setting goals for PA behaviour change.

##### Patient toolkit component

The patient toolkit was designed for delivery by healthcare professionals within routine consultations to assist patients with type 2 diabetes to develop the knowledge and skills for effective use of PA in the self-management of their condition. The toolkit included (1) a discussion card comprised of a 7-day recall used to gauge current levels and patterns of PA, a decisional balance aid [42] to facilitate discussions about pros and cons for changing PA behaviour versus PA behaviour remaining the same, and readiness rulers to gauge the importance of PA and confidence for increasing PA [40]; (2) a booklet to support goal setting, action planning and barrier identification/problem-solving [14]; (3) activity planners, trackers and a pedometer (SW200, Yamax Corporation, Tokyo, Japan) to facilitate time management and self-monitoring of PA; (4) a DVD to promote observational learning and increase self-efficacy; (5) a record of progress pad to guide healthcare professionals through the process of intervention delivery and a mechanism for feedback provision to patients; (6) a motivational postcard mailed out to patients at the 3-month time point to prompt PA behaviour; and (7) and a Diabetes UK leaflet entitled ‘Keeping active: an essential part of managing diabetes’ [43]. Table 2 provides a synopsis of the patient toolkit resources and how their form and information content mapped onto theoretical constructs of TPB and SCT.

**Table 2:** Components of the patient toolkit and their relationship to constructs within the theory of planned behaviour and social cognitive theory

Intervention component	Form and information content	Theoretical constructs	Behaviour change techniques	Evidence source
Discussion card	Assessment of PA behaviour using a 7-day recall A decisional balance aid to assess the pros versus the cons for changing PA behaviour Rulers assessing importance and confidence for change	Attitudes/beliefs (TPB) Forethought (SCT) Intention (TPB) Perceived behavioural control (TPB) and self-efficacy (TPB)	Provide feedback on performance (19) Time management (38)	Exploratory work Systematic review [14]
Booklet	Support to select an appropriate PA/exercise, set PA goals, consider means of social support, identify barriers/problem solve, set short- and long-term goals, plan activity, self-monitor activity, prevent relapse	Forethought (SCT) Subjective norms (TPB) Intention (TPB) Self-regulation (SCT) Perceived behavioural control (TPB) and self-efficacy (TPB)	Goal setting behaviour (5) Social support (29) Barrier identification/problem-solving (8) Time management (38) Self-monitoring (16) Action planning (7)	Systematic review [14, 24, 25]
Activity Planners/Trackers	Means to plan and monitor PA/exercise	Self-regulation (SCT)	Action planning (7) Self-monitoring (16)	Systematic review [24, 25]
DVD	Video recordings of adults with type 2 diabetes engaging in PA/exercise and sharing their stories	Symbolising (SCT) Attitudes/beliefs (TPB) Observational learning (SCT) Subjective norms (TPB) Perceived behavioural control (TPB) and self-efficacy (SCT)	Providing information on the consequences of behaviour to the individual (2)	Exploratory work
Pedometer	Device to monitor the number of steps taken each day	Self-regulation (SCT)	Self-monitoring (16)	Systematic review [24, 25, 27]
Record of progress pad	Record of readiness ruler outcomes, short- and long-term PA/exercise goals, social support, potential barriers and ways to overcome them, self-monitoring method adopted and activities of choice. Provides a mechanism for provision of feedback and an opportunity to monitor progress and recap during subsequent sessions	Perceived behavioural control (TPB) and self-efficacy (SCT) Intention (TPB) Self-regulation (SCT)	Goal setting behaviour (5) Barrier identification/problem-solving (8) Goal setting behaviour (5) Self-monitoring (16) Feedback on performance *(19)*	Systematic review [14, 24, 25]
Diabetes UK leaflet	Leaflet entitled, keeping active: an essential part of managing diabetes	Attitudes/beliefs (TPB) Perceived behavioural control (TPB) and self-efficacy (SCT) Intention (TPB)	Provide information on the consequences of behaviour in general (1)	Exploratory work

The numbers in parentheses correspond to the BCT number in the CALO-RE taxonomy

MaMT2D Movement as Medicine for Type 2 Diabetes, TPB theory of planned behaviour, SCT social cognitive theory

Following an initial discussion of current PA levels with patients, healthcare professionals would select the most appropriate behaviour change techniques (from those presented in modules 6 and 7 of the online programme) that would allow them to tailor the delivery of the patient toolkit in accordance with an individual patient’s needs. A number of strategies are included to target motivation and confidence to support intention formation (e.g. provision of positive feedback on current levels of PA, a discussion of *pros versus cons* for increasing PA versus activity levels staying the same, prompting focus on past success). Once a patient forms an intention to change their level of PA, a number of strategies are included in the process to help them to translate an intention into action. These include self-regulation strategies such as goal setting, action planning and self-monitoring. In addition, a range of behaviour change counselling skills based on the principles of motivational interviewing [44] was integrated into the online programme to engage patients in collaborative decision-making with healthcare professionals.

### Stage 3: assessing usability of the alpha prototype PA behaviour intervention in primary care

*Aims*: To assess usability of the alpha prototype intervention from the perspective of healthcare professionals, data managers and adults with type 2 diabetes from one primary care practice.

#### Methods: primary care staff

One diabetes nurse practitioner, two practice nurses and three data/IT managers from one primary care practice were given access to the alpha prototype PA behaviour intervention. The aim was to elicit their views on the relevance of the content and general usability of both the online training programme and the patient toolkit components. Although healthcare professionals were not required to use the materials during consultations with patients, they were asked to consider while reviewing the intervention components whether they felt they would work in practice and to report any barriers or enabling factors to implementation. Data/IT managers were asked to review the content of the online training programme and to specifically identify any potential barriers to implementation with their existing information technology infrastructure.

Each usability testing participant completed a structured questionnaire asking specific questions about each module in the online training programme (e.g. module 1: does this module provide sufficient information about the purpose of the programme?; how could this module be improved?). Participants were also asked to rate the intervention on a scale of 1 (very dissatisfied) to 10 (completely satisfied). Following a review of the responses from the questionnaire, informal follow-up discussions were carried out with the three healthcare professionals and one data/IT manager (two IT managers opted out of the informal discussions as they did not feel they could comment upon the use of the intervention during routine consultations) to explore in more depth the points raised in relation to their expectations and barriers/facilitators to implementation.

#### Results of usability testing with primary care staff

Minor technical problems with the video footage (e.g. initial freezing of footage and incompatibility with early versions of specific Internet browsers that were installed onto practice computers) and interactive components of the online training programme (e.g. issues with functionality) were detected and subsequently resolved. Further information content was requested on appropriate advice to provide to people with comorbidities (e.g. arthritis) when planning to undertake PA. This additional information was subsequently added to module 5 of the online training programme. Comments from primary care staff and responses to the structured questionnaire (median satisfaction score was 8.0; IQR 2.0) indicated that satisfaction with both intervention components was high, and they emphasised that they would use the intervention and recommend it to colleagues. When asked how they would use the programme, they suggested that they would complete it in full initially but would like to return to relevant sections if and when required. Therefore, the need to have continuous access to the online programme was emphasised.

#### Methods: usability testing with patients

Structured interviews were also conducted with 13 adults with type 2 diabetes attending primary care diabetes clinics at one primary care practice (*n* = 8 males, *n* = 5 females, mean age = 58 years (SD = 14), mean time since diagnosis = 5 years (SD = 5)). Patients were approached following their diabetes review appointment with a healthcare professional to assess the face and content validity of the patient toolkit. During the interview, patients were taken through a typical scenario with the toolkit to identify any content, design or usability issues. A structured questionnaire requested information about their views on each aspect of the toolkit, for example, ‘what, if anything would make the activity planners easier to use?’

#### Results of usability testing with patients

Although no significant issues were identified with the content or usability of the patient toolkit, patients suggested that the original ‘intervention task card’ design should be redeveloped into a booklet format. They also commented that pictures of physical activities should be added to the reverse side of the toolkit case and that the booklet should include provision to record appointment dates and times, including space to make a note of any questions to ask during subsequent appointments. Patients indicated that they would use the materials with the support of a healthcare professional as a mechanism to receive feedback and would be interested in taking part in the programme. Mirroring the findings of the initial exploratory work, the latter was driven by a desire to learn more about PA, as the majority of patients reported that they did not recall having been advised specifically about PA to manage their diabetes or offered support to increase their levels of PA to enable them to self-manage their diabetes.

### Stage 4: an open pilot study to assess acceptability and feasibility in the primary care setting and to optimise intervention components

Based on findings from the usability testing with healthcare professionals and patients, a beta prototype of *Movement as Medicine for Type 2 Diabetes* was developed for use in a subsequent open pilot study in the primary care setting.

#### Design

A single-arm open pilot study with baseline and follow-up assessment at 1 month was conducted in two primary practices in North East England. An open pilot study design was selected to enable a preliminary assessment of acceptability, feasibility and fidelity of delivery and to facilitate systematic adaptations and refinements of the intervention while being used in a real-life setting [45]. Intervention components were optimised throughout a 1-month intervention period (baseline and 1 month diabetes review appointments) by monitoring access and use of the online training programme by healthcare professionals, theoretical domain framework informed interviews with primary healthcare professionals and patients, and analysis of video recordings of consultations to investigate fidelity of intervention delivery by healthcare professionals. By systematically collecting information and refining the intervention, the aim was to optimise intervention components and increase acceptability, feasibility and fidelity of delivery of the intervention for use in the primary care setting ahead of a planned pilot RCT.

#### Participants

**Fig. 2:**
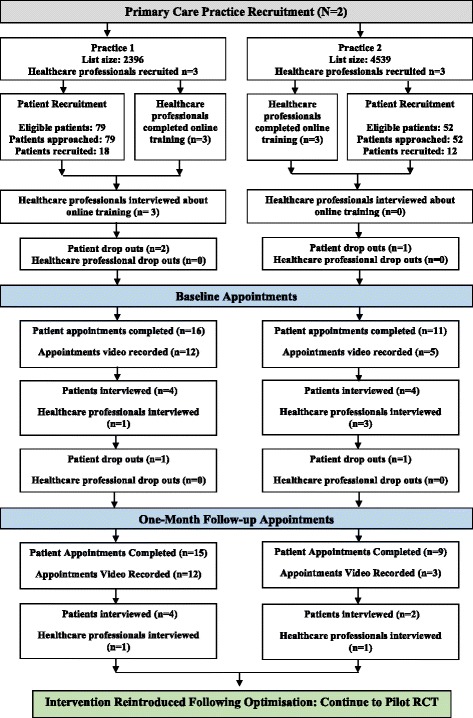
Summary of practice, healthcare professional, patient recruitment and data collection activity

Two primary care practices agreed to participate in the pilot study who allocated six primary healthcare professionals (two GPs, three practice nurses, one healthcare assistant) to participate in the study (see Fig. 2). One GP and one practice nurse were specialists in diabetes. All six healthcare professionals were female, aged 40 to 55 years and had not previously received training on health psychology theory and principles of health behaviour change in general or specific to PA. However, two healthcare professionals reported having attended a workshop promoting the use of PA in the primary care setting. None of the participating healthcare professionals had previously been involved in diabetes or PA research.

Out of 131 eligible patients invited to participate in the open pilot study (79 and 52 from practices 1 and 2, respectively), 30 (23 %) adults with non-insulin-dependent type 2 diabetes were recruited (*n* = 18 from practice 1 and *n* = 12 from practice 2). This sample size was consistent with the standard guidance relating to sample size for pilot studies [45, 46]. Patients were aged between 46 and 88 years (mean 68.9; SD 10.6); 60 % were male (M/F, 18/12); time since diagnosis of type 2 diabetes was between 2 and 28 years (median 4.5; IQR 5.0); and 21 patients managed their diabetes with oral medication and 9 with diet. Patients managed with insulin were excluded due to increased risk of hypoglycaemia as a result of increased levels of PA.

Consent was obtained from primary healthcare professionals to complete the online training programme and to subsequently deliver the intervention toolkit to patients recruited to the study. Separate consent was given by healthcare professionals and patients for video recording of the consultations to assess fidelity of delivery of behaviour change techniques in practice and to take part in one or more interviews with a researcher to identify barriers and enabling factors to implementation of the intervention.

#### Data collection

Healthcare professionals were emailed a unique login ID and link to the online training programme that had to be completed before the delivery of the intervention to participating patients. They were given 4 weeks to complete the training programme. Completion of all eight modules of the programme was confirmed by generation of an electronic personalised certificate. Usage of the online training programme was monitored to establish whether healthcare professionals completed the training programme within the required time period (i.e. 4 weeks). Monitoring was also used to establish whether the programme was revisited following initial completion and feasible for completion within the primary care working environment.

Healthcare professionals were interviewed following completion of the online training programme and again following the delivery of the intervention during baseline and 1-month patient appointments. Interviews focused specifically on the acceptability and feasibility of the intervention, including ways to increase implementation and refine study processes and procedures. The interview topic guides were developed using the Theoretical Domains Framework (TDF) [47]. The TDF is the result of a multi-disciplinary expert consensus approach that aimed to organise and simplify the theoretical literature on behaviour change by reviewing 33 behavioural theories. The 128 key constructs were organised into broad theoretical domains based on commonalities [48]. The TDF consists of 14 theory domains each providing broad explanations of why a behaviour may or may not have been performed: knowledge; skills; professional role and identity; beliefs about capabilities; optimism; beliefs about consequences; reinforcement; intentions; goals; memory, attention and decision processes; environmental context and resources; social influences; emotions; and behavioural regulation [47]. This approach enabled a range of personal, professional and environmental challenges to implementation of the intervention to be explored.

Although the TDF is not a theory, it enables the interview findings to be linked back to theory to facilitate an understanding of the behaviour change processes that were inhibitors or drivers of implementation. Theory-driven refinements could then be made to optimise the intervention content and processes (i.e. domain-specific beliefs informed changes/improvements to intervention components) that were congruent with the TPB and SCT used to develop the intervention components (described in stages 1 to 3). It was used to identify domain-specific issues with the online training programme and delivery of the patient toolkit (e.g. knowledge/skills/support gaps).

Acceptability and feasibility of the patient toolkit from the perspective of patients were assessed using TDF-informed semi-structured interviews to establish comprehension, acceptability and feasibility of the toolkit and to detect any domain-specific issues (e.g. information/support gaps). Interviews were conducted with patients immediately following their diabetes review appointments incorporating the intervention at baseline and 1-month follow-up.

The TDF was used to identify domain-specific beliefs likely to positively or negatively impact on acceptability and feasibility of the intervention in the primary care setting and to inform intervention optimisation.

#### Assessing fidelity of delivery of intervention components by healthcare professionals

Fidelity of delivery of the intervention components was assessed to facilitate optimisation of the intervention (i.e. to identify specific intervention components that did not translate well into practice) and to enable adaptations to be made to the intervention to improve implementation [49]. Failure to assess fidelity of delivery of intervention components could potentially lead to false conclusions regarding the effectiveness or ineffectiveness of interventions. Effectiveness may be due to non-intervention factors and failure of interventionists to deliver intervention components in accordance with the protocol. Fidelity of delivery assessment is critically important because there is a risk that ineffective interventions are adopted into clinical settings in place of potentially effective interventions [49, 50]. In order to assess whether the patient toolkit intervention could be consistently and faithfully delivered in the primary care setting by healthcare professionals, a fidelity checklist was constructed to assess the presence and absence of specific intervention content (behaviour change techniques) being delivered in accordance with intervention protocol. Where intervention components were more frequently omitted, adaptations to the intervention were made to improve fidelity of delivery following completion of the 1-month appointments.

#### Data analyses

Descriptive statistics (median and IQR, minimum and maximum values) were calculated for time spent (in minutes) by healthcare professionals to complete the online training programme and over what period of time (e.g., seven consecutive or non-consecutive days). In addition, data were collected to determine whether healthcare professionals revisited the online training programme post-completion and how much time they spent browsing the programme in minutes (median and IQR, minimum and maximum values). These analyses allowed an assessment of feasibility (i.e. whether healthcare professionals could complete the online training within the time permitted or whether modifications were required) and acceptability (i.e. whether healthcare professionals were willing to use the online training programme and return to the programme throughout the intervention period).

TDF-informed interview transcripts with healthcare professionals (*n* = 9) and patients (*n* = 14) were content analysed [22] independently by the first author (LA) using NVivo 10 [51] and the TDF and its constructs as a coding frame. Twelve of the 23 transcripts were independently coded and content analysed by a second researcher (SJC). The first TDF-informed interview transcript generated was independently coded by both researchers and discussed (to ensure consistency) before coding subsequent transcripts. Regular meetings were held between the two researchers where discrepancies were resolved via discussion. Data saturation was defined in accordance with published research evidence for theory-based interviewing [52].

Video recordings of consultations between healthcare professionals and patients (*N* = 32) were coded by two of the three researchers (LA, SD, KK) using a fidelity checklist to ensure reliability. All three coders had expertise in health behaviour change and experience of using the 40-item taxonomy [23] utilised throughout the intervention development process. While reviewing the video footage of each consultation, two coders independently recorded each intervention component as ‘yes’ (delivered) or ‘no’ (not delivered). Disagreements were resolved via discussion with the third researcher while revisiting video footage where discrepancies existed.

## Results

### Time spent completing the online training programme and modules reviewed post-training by primary healthcare professionals

All six healthcare professionals logged on and accessed the online training programme. They spent a median of 3 h and 35 min browsing the programme up to the point of completion (i.e. when a certificate was generated) over a median period of 5.5 days (see Table 3). The median time spent using the programme post-completion was 58 min over a median period of 4 days.

**Table 3:** Data on time spent browsing the programme (up to completion and post-completion)

	Number of days spent in training	Total hours/minutes	Number of days spent in training post-completion	Total hours/minutes
Min	2.00	00:57	1.00	00:28
Max	9.00	07:02	10.00	06:42
Range	7.00	06:05	9.00	06:13
Median	5.50	03:35	4.00	00:58
IQR	3.25	01:21	5.50	02:33

### Healthcare professional perspectives: identification of domain-specific beliefs that impact upon acceptability and feasibility of the intervention

Barriers to acceptability and feasibility of the intervention from the perspective of healthcare professionals were associated with six theoretical domains. These were memory, attention and decision processes (i.e. difficulties recalling specific components of the intervention); optimism (i.e. that the intervention is unlikely to help change PA behaviour of patients); environmental context and resources (i.e. lack of time to complete training and practice intervention delivery); social influences (i.e. improving diabetes care is considered to be a practice norm and something healthcare professionals are already doing); professional role and identity (i.e. belief that nurses and not GPs should be delivering the intervention); and beliefs about consequences (i.e. the intervention will only work with a minority of patients).

A total of 25 domain-specific beliefs across all 14 TDF domains were found to positively influence acceptability and feasibility of the intervention in the primary care setting. The most frequently reported domains included knowledge (i.e. completing the online training programme has improved knowledge of diabetes and the value of PA for glycaemic control); skills (i.e. the training programme has facilitated acquisition of behaviour change skills); beliefs about capabilities (i.e. practice delivering the intervention to patients will make it easier to use); intentions (i.e. completing the training programme has increased the likelihood that PA will be targeted in future consultations); and optimism (i.e. practice will improve delivery of the intervention during routine consultations and lead to beneficial changes in patient behaviour).

### Patient perspectives: identification of domain-specific beliefs that impact on acceptability and feasibility of the intervention

Barriers to acceptability and feasibility of the intervention from the perspective of patients were associated with three theoretical domains. These were memory, attention and decision processes (i.e. it takes time to fully understand the nature of the intervention and its components); environmental context and resources (i.e. a period of ill health or adverse health events could create barriers to increasing levels of PA); and social influences (i.e. a preference to complete the programme alone/privately without social support).

A total of 40 domain-specific beliefs from across all 14 domains of the TDF were found to impact positively on acceptability and feasibility of the intervention by patients. Ten theoretical domains were most frequently reported: knowledge (i.e. the intervention increased knowledge and awareness of the benefits of PA for management of type 2 diabetes); skills (i.e. the intervention equipped patients with the self-management skills to plan, monitor and overcome barriers to PA); beliefs about capabilities (i.e. increasing the levels of PA was not as difficult as anticipated when using the intervention); beliefs about consequences (i.e. increasing the levels of PA will have important health benefits); optimism (i.e. the intervention will help to increase and maintain the levels of PA); reinforcement (i.e. feedback and social support from healthcare professionals during consultations is an *incentive* to taking part); intentions (i.e. the intentions to attempt to increase the levels of PA have become stronger as a result of the intervention); goals (i.e. setting personal goals is an important aspect of the intervention); social influences (i.e. practical and emotional support from healthcare professionals at the primary care practice is important for maintaining motivation); and behavioural regulation (i.e. self-monitoring activity levels have facilitated an increase in PA).

### Fidelity of intervention delivery by healthcare professionals

Inter-rater reliability using Cohen’s kappa calculated for baseline and 1-month coding of intervention content was 0.60 and 0.55, respectively, indicating moderate agreement.

Assessment of video recordings revealed that the majority of behaviour change techniques and other intervention components (i.e. agenda setting, discussion of pros versus cons for increasing PA, and utilisation of importance and confidence rulers) were delivered faithfully during baseline and 1-month follow-up appointments for the majority of the time. The intervention components frequently not delivered when it would have been appropriate to deliver them included agenda setting; discussion of pros versus cons for increasing PA; barrier identification and problem-solving; prompt focus on past success; prompt rewards contingent on progress and time management. The behaviour change technique review of behavioural goals was, as expected, omitted from all baseline consultations but delivered during 15 of the 17 1-month follow-up consultations. The behaviour change technique prompt generalisation of PA behaviour was not delivered during baseline or 1-month follow-up consultations.

### Patient and healthcare professional perspectives: how the data obtained informed intervention optimisation to improve acceptability and feasibility of the intervention

Analyses of data collected during the open pilot study indicated that the intervention was acceptable and feasible for use with healthcare professionals and patients. The data were subsequently used to inform a number of refinements to intervention components and processes to improve acceptability, feasibility and fidelity of delivery during the pilot RCT. An overview of these refinements to the intervention components is presented in Table 4.

**Table 4:** An overview of modifications made to the Movement as Medicine for Type 2 Diabetes multifaceted intervention

Revision	Source of data that informed revision	Justification for revision
Removal of the behaviour change technique ‘rewards contingent on progress towards behaviour’	Semi-structured interviews with healthcare professionals and patients and video recordings of consultations	Several attempts were made by healthcare professionals to deliver the technique; however, faithful delivery and quality of delivery emerged as ongoing issues. Healthcare professionals reported the technique as ‘uncomfortable’ to deliver and felt it was not well received by patients. Patients reported the technique as ‘unnecessary’ and considered positive feedback from healthcare professionals a sufficient reward. The intervention contains a number of other self-regulatory behaviour change techniques; therefore, the balance of motivational and volitional techniques was maintained
Inclusion of explicit feedback on outcomes of behaviour	Semi-structured interviews with patients	Patients requested a formal record of weight, waist circumference, blood pressure and glycaemic control (HbA1c) to allow them to monitor their behavioural progress against their own anthropometric and clinical outcomes. The ‘record of progress pad’ was subsequently redesigned to allow provision for this information. Removal of the technique ‘rewards’ and inclusion of ‘feedback on outcomes of behaviour’ ensured that the balance of motivational and volitional techniques was maintained
Redesign of the record of progress pad	Semi-structured interviews with healthcare professionals and patients	Healthcare professionals reported that there was insufficient space within version 1 of the record of progress pad to record PA goals and plans. Patients requested provision to record and monitor outcomes of behaviour. The record of progress pad was subsequently revised/optimised and reintroduced
Inclusion of an intervention component checklist	Semi-structured interviews with healthcare professionals	Healthcare professionals requested a paper-based checklist of intervention components/techniques available to them via the Movement as Medicine for Type 2 Diabetes intervention. They reported that this ‘visual aid’ would reduce cognitive burden, prompt use of specific techniques and thus increase the likelihood of implementation of intervention components
Insertion of an online contents page to direct users to descriptions of intervention components	Semi-structured interviews with healthcare professionals	An additional page was inserted into the online training programme to direct healthcare professionals to descriptions of each intervention component and examples of how each component could/should be used. The aim was to (1) increase fidelity of delivery of intervention components overall and to specifically target those that were frequently not delivered and (2) to increase quality of delivery
Insertion of additional online content to promote delivery of specific BCTs	Video recordings of consultations	Additional written information was inserted online to prompt delivery of barrier identification/problem-solving (i.e. to prompt use of problem-solving specifically) and time management

**Fig. 3:**
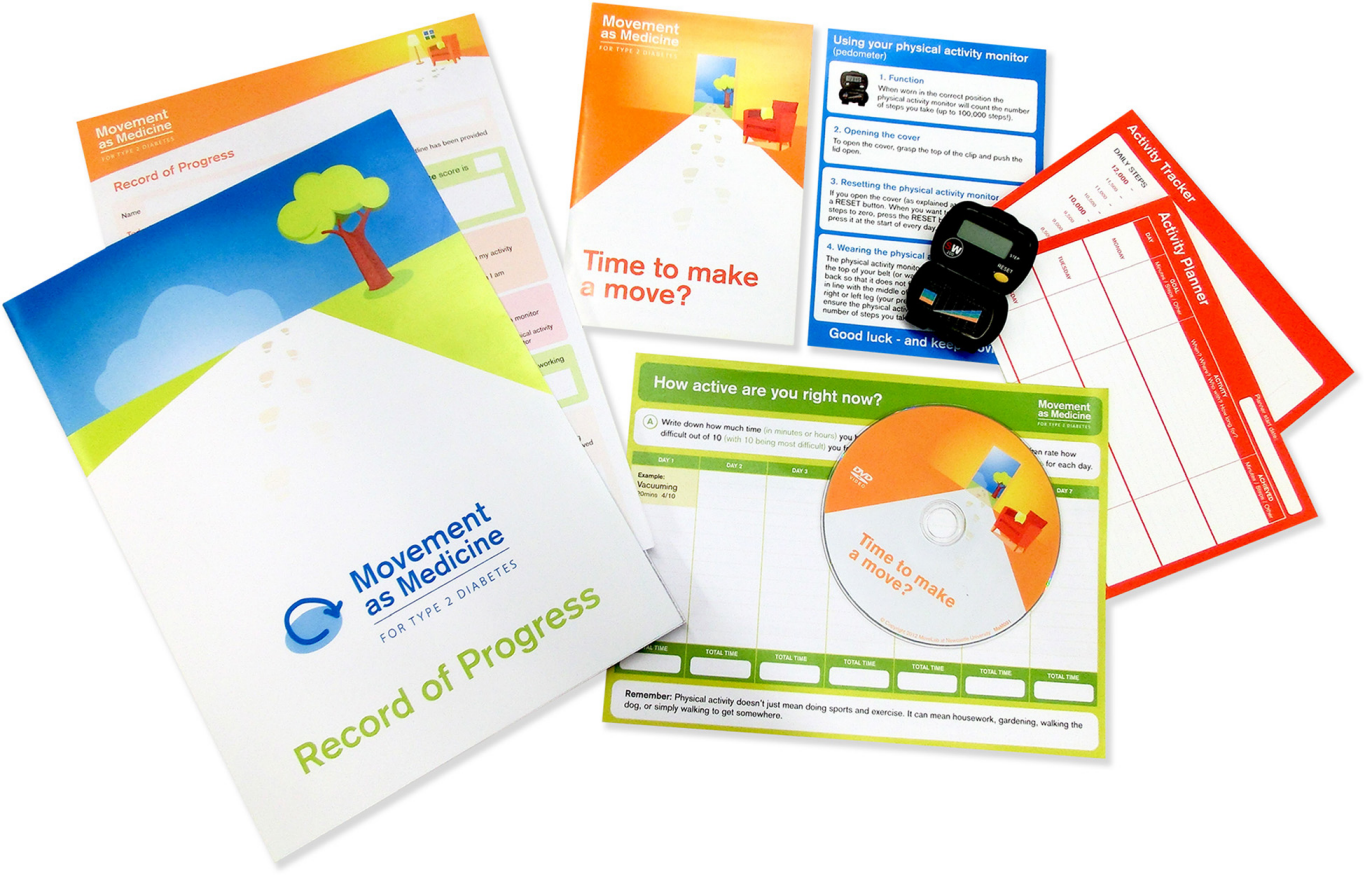
Image of the Movement as Medicine for Type 2 Diabetes intervention toolkit

Healthcare professionals and patients in the open pilot study continued onto the pilot RCT. Healthcare professionals were asked to revisit the training programme to view content added as an outcome of the open pilot study and deliver the intervention to patients during the 6- and 12-month diabetes review appointments (i.e. 6- and 12-month follow-up). An illustration of the Movement as Medicine for Type 2 Diabetes intervention, following revisions, is presented in Fig. 3.

## Discussion

This paper presents a systematic, theory and evidence-informed approach to developing a multifaceted behavioural intervention for use by healthcare professionals (GPs, practice nurses and healthcare assistants) to promote PA as an effective component of self-management amongst adults with type 2 diabetes in the primary care setting.

The systematic intervention development process addressed an important criticism of previous behaviour change interventions targeting PA—the absence of a detailed description of the rationale for selecting theory, information content, duration and intensity of interventions that prohibits replication [23]. Adherence to the MRC framework to guide the development of Movement as Medicine for Type 2 Diabetes provides an audit trail for the provenance of the intervention’s facets (online training programme and patient toolkit). Moreover, meaningful involvement of healthcare professionals and patients in an iterative and co-development process provides evidence that Movement as Medicine for Type 2 Diabetes (mode, form and information content) is sensitive to their preferences and the context of intervention delivery, which can enhance implementation and uptake in the clinical setting. A further strength of our approach was the use of a taxonomy [23] to ensure that behaviour change techniques were defined consistently throughout the intervention development process.

The findings of the systematic review and meta-analyses were instrumental in establishing a robust rationale for the development of a theory-informed behaviour change intervention targeting PA of adults with type 2 diabetes in community and clinical settings with the use of behavioural strategies. Furthermore, it highlighted the importance of providing evidence-based skills development training to healthcare professionals to facilitate delivery of intervention content.

While a prodigious amount of work has gone into elucidating a taxonomy of behaviour change techniques, there has been less attention directed towards effective strategies to provide interventionists with the knowledge and skills required to deliver theory-linked behaviour change techniques as described in the taxonomy. The current study adds to the evidence base by providing a theory-based means of operationalising the prerequisite of behaviour change skills required and conveying this audiovisually via an online intervention (training programme) to support ‘vicarious skills development’. Furthermore, the online training intervention programme provides an important proof of concept for the delivery of behaviour change techniques by non-psychologists, which reflects one of the key aims of the behaviour change technique taxonomy [23].

The feasibility and acceptability of the online training intervention programme for improving the knowledge and skills of primary healthcare professionals were consistent with a Cochrane review published in 2013 on a digital/Internet-based PA behaviour change interventions [53]. Another systematic review published in 2010 of Internet-based health behaviour change interventions reported that such interventions had small but significant effects on health behaviour, although interventions underpinned by the TPB and those incorporating more behaviour change techniques and using additional methods of communicating (e.g. text messaging) were reported to have larger effects [53]. These findings support the decision to utilise the TPB to underpin the intervention and add to the evidence base for Internet-based interventions to support professional behaviour change [54]. However, in acknowledgment of the limitations of the TPB, specifically in relation to its utility for developing intervention content [55], we engaged healthcare professionals and patients in an iterative development process. This helped to identify acceptable and feasible ways of operationalising theoretical constructs from the TPB and SCT (and specific BCTs) to ensure that the intervention mode, form and content were optimally sensitive to the study context.

An assessment of fidelity of intervention delivery by healthcare professionals addressed an important methodological issue in the behaviour change literature. That is whether intervention components can be delivered faithfully by interventionists within the constraints of the target setting. We were able to demonstrate that ‘enactment’ of intervention knowledge and skills (i.e. specific behaviour change techniques) in the primary care setting can be attributed to the completion of the evidence-based online training programme, described as the theoretically expected treatment effects in the MRC framework [18, 23].

The use of an open pilot study design enabled the gathering of feedback from healthcare professionals and patients throughout an active intervention period [23]. While early development work is essential to make important decisions about intervention content and mode of delivery, often, implementation issues cannot be identified until the intervention is utilised in practice. Therefore, the open pilot study design allowed the intervention to be ‘tested’ and refined ahead of a planned pilot RCT involving a larger number of primary care practices and participants. Several issues emerged that were later targeted by refinements to the intervention. These included failure to deliver intervention components when it would have been appropriate to deliver them (e.g. use of problem-solving when patients report barriers to undertaking physical activity); behaviour change techniques not delivered because healthcare professionals felt they were not relevant to the individual patients’ circumstances (e.g. omission of the behaviour change technique *time management* when the patient is retired); and quality of delivery (i.e. not delivering behaviour change techniques in accordance with their published definitions and patient needs, preferences and circumstances). The latter made fidelity of delivery assessment difficult.

The TDF approach has been used to understand patient and healthcare professional behaviour and intervention implementation challenges in a variety of contexts such as witness response at acute onset of stroke [56]; implementation of a complex intervention for acute low back pain management in primary care [57]; hand hygiene behaviours [58]; implementation of family intervention recommendations within the NICE guideline for schizophrenia [59]; and clinicians’ behaviour about preoperative test ordering for anaesthesia management [60] and blood transfusion [61]. However, despite wide and successful use, there are several potential limitations of the TDF approach that should be acknowledged. Firstly, it is a descriptive framework not a theory; therefore, relationships between domains cannot be specified. Secondly, the TDF is frequently used in interview studies; however, inter-coder agreement can be low [62]. This highlights the issue that it can be difficult to recognise the boundaries between the domains when using the TDF as a coding framework [62].

Although the systematic intervention development process presented has several strengths, the limitations should be acknowledged. The early exploratory work was based on a very select and small sample of healthcare professionals; therefore, we cannot be certain that the findings were representative. Behaviour change techniques identified by the systematic review [14] were based on univariate analyses and only 25 out of 40 were used across the 17 included RCTs. Therefore, it is yet to be determined whether the behaviour change techniques omitted could have further optimised the intervention. Moreover, specific clusters of BCTs may be or less effective at different junctures in the intervention process.

Further avenues of research, which could augment the information content of Movement as Medication, include the provision of ‘personalised’ information to individuals with type 2 diabetes on the likely absolute benefits of increasing their levels of PA. However, there has been limited research looking into the impact of numerical and graphical information on risk in the context of type 2 diabetes. The @Risk trial investigated the utility of theory-based methods of conveying absolute 10-year risk of cardiovascular disease to patients with type 2 diabetes [63]. This trial found a positive effect on the accuracy of patients’ estimates of cardiovascular risk perception (agreement between the patient’s UK Prospective Diabetes Study risk engine score for cardiovascular disease and the patient’s perceived risk) at 2 weeks, but not 12 weeks, with no significant impact on attitudes and intentions to make lifestyle behaviour changes. Further research is warranted to establish whether evidence-based information that transparently communicates the absolute risk reduction/benefit of developing complications and/or progressing to insulin treatment as a function of incremental changes to patients’ current levels of PA could further support the formation of positive outcome expectancies (in both patients and healthcare professionals). In addition, an assessment is needed as to whether this could serve as a further mechanism for augmenting the BCT’s ‘provide information on the consequences of behaviour to the individual’ and supporting collaborative discussions with healthcare professionals about self-management strategies for increasing PA in the primary care setting.

Future research is needed to explore the impact of providing individualised feedback on performance to healthcare professionals as part of an enhanced training programme to establish any additional benefit in terms of fidelity of delivery and successful implementation of behavioural interventions in the primary care setting. The Movement as Medicine Intervention could also be provided as a compliment to existing programmes such as DESMOND and X-PERT by providing healthcare professionals with the skills to provide ongoing support.

With the recent inception of Clinical Commissioning Groups (CCGs) in the UK, there is arguably a greater need to demonstrate cost-effectiveness as well as clinical effectiveness. The former will need to be a core component of future randomised studies. This will elucidate any improvement in outcomes as a function of cost savings to the UK National Health Service and as a function of cost of the intervention per patient against the cost savings of the reduced diabetes-related morbidity and mortality.

Threats to continued uptake of the intervention over time should be considered. These include the need for updating the Movement as Medicine for Type 2 Diabetes intervention over time as new evidence emerges on the effectiveness of behaviour change models/theory, theory-linked behaviour change techniques and types of PA and exercise, including sedentary behaviour [64]. Otherwise, after a relatively short period of time, the intervention may be viewed as *out of date* and that would impact negatively on uptake rates by healthcare professionals over time (and ultimately prevent patients from receiving the support they need to increase their PA behaviour). Furthermore, information computer technology is progressing rapidly, and to maintain uptake rates, there is also need to update and maintain the online training programme to ensure compatibility with primary care systems.

## Conclusions

Despite PA being widely considered to be a cornerstone of diabetes care, rarely are healthcare professionals trained to provide support to adults with type 2 diabetes to become more physically active. In accordance with the MRC framework for development and evaluation of complex interventions [18], we have described the systematic development of a multifaceted PA behavioural intervention with reference to the pre-clinical (theoretical), modelling (phase I) and exploratory trial (phase II) phases. All phases contributed meaningfully to the intervention development process. In particular, the mixed methods open pilot study with assessment of fidelity was most useful for the following reasons: increased confidence that the intervention could feasible be delivered as specified, allowed the intervention to be optimised ahead of a planned pilot RCT and provided invaluable insights into the implementation of the intervention into routine practice. Following optimisation and a preliminary positive assessment of acceptability, feasibility and fidelity at 1-month follow-up with healthcare professionals and patients, future research will compare the Movement as Medicine for Type 2 Diabetes multifaceted PA behavioural intervention to standard clinical care in a pilot RCT [21].

## Abbreviations

GP, general practitioner; PA, physical activity; RCT, randomised controlled trial; MRC, Medical Research Council; HbA1c, glycated hemoglobin A1c; TDF, Theoretical Domains Framework; TPB, theory of planned behaviour; SCT, social cognitive theory

## Additional files

Additional file 1: Interactive workshop materials (‘Circle of influence’ worksheet). (PDF 36 kb)

Additional file 2: Interactive workshop materials (‘My diabetes story’ worksheet). (PDF 34 kb)

Additional file 3: Interactive workshop materials (‘Good healthcare professional’ worksheet). (PDF 24 kb)
