# Supplementary material for: Systematic development of a theory-informed multifaceted behavioural intervention to increase physical activity of adults with type 2 diabetes in routine primary care: Movement as Medicine for Type 2 Diabetes
Source: Implement Sci. 2016 Jul 19;11:99. doi: 10.1186/s13012-016-0459-6 (PMC4950706; doi:10.1186/s13012-016-0459-6)

Type 2 Diabetes - Circle of Influence

Patient

Your name

Where you live

Instructions

Write the names or numbers of the following people and groups on the rings of the Circle of Influence. The closer they are to YOU, the MORE IMPORTANT they are to your successful diabetes management. Leave out any people or groups you have not encountered. Add in any people that we've missed out.

People and groups involved in diabetes management:

1. Your GP
2. GP Practice Nurse
3. Phlebotomist *Blood samples*
4. Optometrist *Eye screening*
5. Podiatrist *Foot care*
6. Dietician
7. Pharmacist
8. DESMOND Programme
9. Endocrinologist *Hormone specialist*
10. Diabetes UK Support Group
11. Psychologist
12. Diabetes Specialist Nurse
13. Diabetes Research Doctor
14. Consultant Diabetologist
15. Physiotherapist
16. Exercise Practitioner

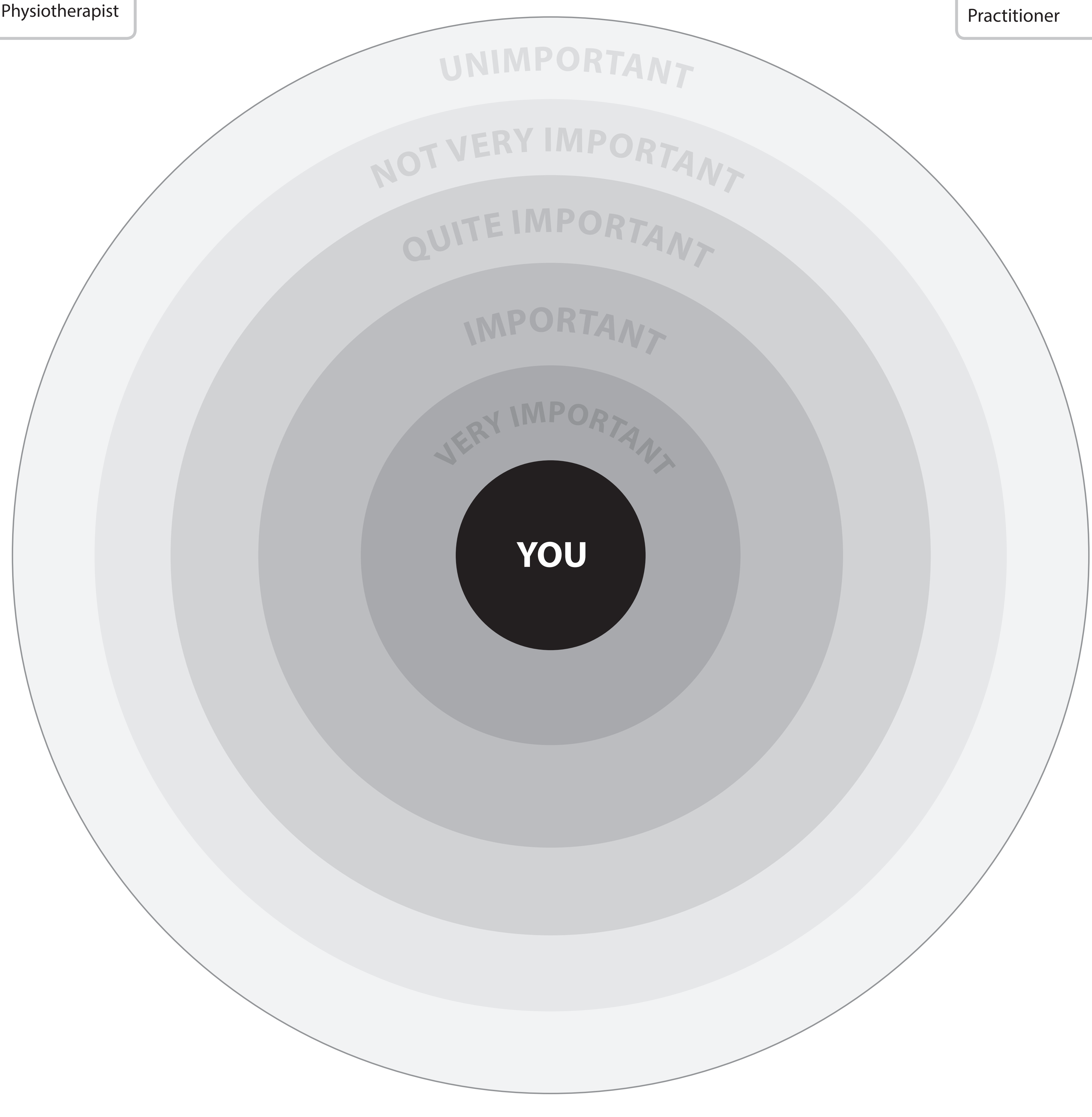

Supplement: Supplementary file 1 — Interactive workshop materials (‘Circle of influence’ worksheet). (PDF 36 kb) [file 13012_2016_459_MOESM1_ESM.pdf]
