# Supplementary material for: Systematic development of a theory-informed multifaceted behavioural intervention to increase physical activity of adults with type 2 diabetes in routine primary care: Movement as Medicine for Type 2 Diabetes
Source: Implement Sci. 2016 Jul 19;11:99. doi: 10.1186/s13012-016-0459-6 (PMC4950706; doi:10.1186/s13012-016-0459-6)
Supplement: Supplementary file 2 — Interactive workshop materials (‘My diabetes story’ worksheet). (PDF 34 kb) [file 13012_2016_459_MOESM2_ESM.pdf]

Type 2 Diabetes - Your Story

Patient

About You

Please note: You can leave PART OR ALL of this section blank if you want. The information you provide will still be useful if it's anonymous. If you do provide contact details, then we may contact you to talk further about your experiences of Type 2 Diabetes.

Your name ____________________ **Where you live** ____________________

Phone number ____________________ **Email Address** ____________________

Diabetes Diagnosis

When were you first diagnosed? ____________________

How did you feel when you were diagnosed?
(circle the word that best describes your reaction)

Relieved *Angry* *Scared* *Surprised* *Well Prepared* *Confused*

What was your doctor's first advice to you? ____________________

What was your biggest worry about diabetes? ____________________

Living with Diabetes

How do you think you are managing your diabetes?
(circle the word that best describes your situation)

Very Well *Quite Well* *Just Coping* *Not Coping At All*

What is your current treatment regime? ____________________

What's the WORST thing about diabetes? ____________________

What's the BEST thing, or the 'silver lining'? ____________________

What ONE THING would you say to someone who's just been diagnosed?

____

How much of your life is about your diabetes?
(fill in the bar to indicate a percentage)

0%	10%	20%	30%	40%	50%	60%	70%	80%	90%	100%

Active living

How active is your lifestyle?
(circle the word that best describes your lifestyle)

Very Active *Quite Active* *Not Very Active* *Not Active At All*

How important do you think activity & exercise are to good diabetes management?
(circle the word that best describes your opinion)

Very Important *Quite Important* *Not Very Important* *Unimportant*

What advice has your GP given you about exercise? ____________________

Have you become more active since your diagnosis? ____________________

What ONE THING stops you being more active? ____________________

What ONE THING would help you be more active? ____________________
