# Supplementary material for: Systematic development of a theory-informed multifaceted behavioural intervention to increase physical activity of adults with type 2 diabetes in routine primary care: Movement as Medicine for Type 2 Diabetes
Source: Implement Sci. 2016 Jul 19;11:99. doi: 10.1186/s13012-016-0459-6 (PMC4950706; doi:10.1186/s13012-016-0459-6)

Type 2 Diabetes - What is a great Health Professional like?

Patient

Instructions

Write the things that make a GREAT Health Professional onto the silhouette below.

WHAT DO THEY LOOK LIKE?

HOW OLD ARE THEY?

WHAT ARE THEY WEARING?

HOW DO THEY TREAT YOU?

WHAT DO THEY SAY?

HOW DO THEY GET RESULTS?

DO THEY TELL YOU WHAT YOU WANT TO HEAR?

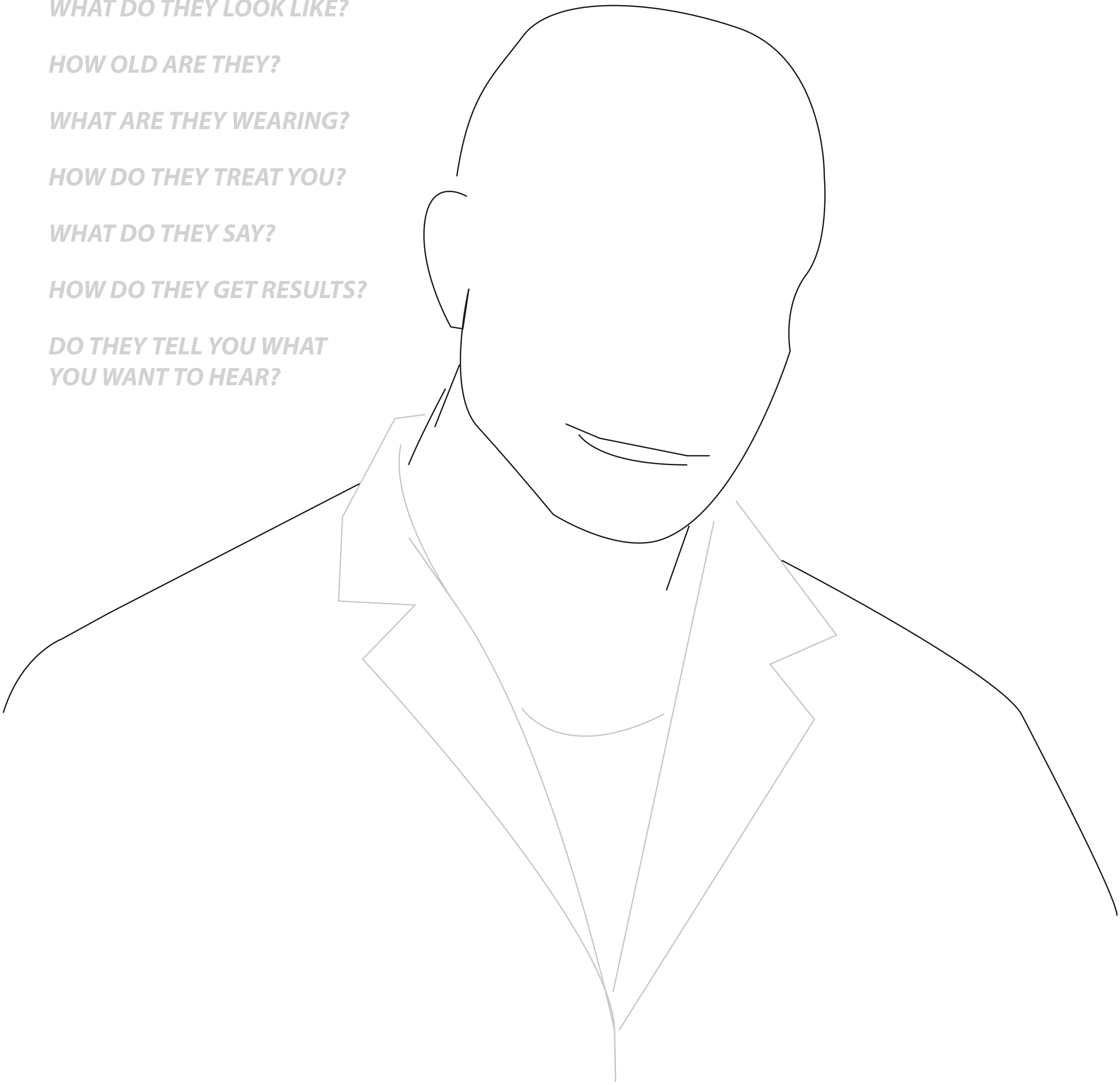

Supplement: Supplementary file 3 — Interactive workshop materials (‘Good healthcare professional’ worksheet). (PDF 24 kb) [file 13012_2016_459_MOESM3_ESM.pdf]
